# The Potential Role of Intestinal Microbiota on the Intestine-Protective and Lipid-Lowering Effects of Berberine in Zebrafish (*Danio rerio*) Under High-Lipid Stress

**DOI:** 10.3390/metabo15020118

**Published:** 2025-02-11

**Authors:** Chang Gao, Heng Wang, Xuan Xue, Lishun Qi, Yanfeng Lin, Lei Wang

**Affiliations:** 1 Collaborative Innovation Center of Recovery and Reconstruction of Degraded Ecosystem in Wanjiang Basin Co-Founded by Anhui Province and Ministry of Education, Anhui Normal University, Wuhu 241002, China; gaochang000610@163.com (C.G.); wangneigejiayuan@163.com (H.W.); 13856855185@163.com (X.X.); lishunqi2005@163.com (L.Q.); 2 Fishery Bureau of Xiuning County, Huangshan 245400, China; 3 Provincial Key Laboratory of Biotic Environment and Ecological Safety in Anhui, Wuhu 241002, China

**Keywords:** berberine, high-lipid diet, lipid lowering, intestinal microbiota, antibiotic

## Abstract

**Background:** Berberine has extremely low oral bioavailability, but shows a potent lipid-lowering effect, indicating its potential role in regulating intestinal microbiota, which has not been investigated. **Methods:** In the present study, five experimental diets, a control diet (Con), a high-lipid diet (HL), and high-lipid·diets·supplemented with an antibiotic cocktail (HLA), berberine (HLB), or both (HLAB) were fed to zebrafish (*Danio rerio*) for 30 days. **Results:** The HLB group showed significantly greater weight gain and feed intake than the HLA and other groups, respectively (*p* < 0.05). Hepatic triglyceride (TG) and total cholesterol (TC) levels, lipogenesis, and proinflammatory cytokine gene expression were significantly upregulated by the high-lipid diet, but significantly downregulated by berberine supplementation. Conversely, the expression levels of intestinal and/or hepatic farnesoid X receptor (*fxr*), Takeda G protein-coupled receptor 5 (*tgr5*), lipolysis genes, and zonula occludens 1 (*zo1*) exhibited the opposite trend. Compared with the HLB group, the HLAB group displayed significantly greater hepatic TG content and proinflammatory cytokine expression, but significantly lower intestinal bile salt hydrolase (BSH) activity and intestinal and/or hepatic *fxr* and *tgr5* expression levels. The HL treatment decreased the abundance of certain probiotic bacteria (e.g., *Microbacterium*, *Cetobacterium*, and *Gemmobacter*) and significantly increased the pathways involved in cytochrome P450, p53 signaling, and ATP-binding cassette (ABC) transporters. The HLB group increased some probiotic bacteria abundance, particularly BSH-producing bacteria (e.g., *Escherichia Shigella*). Compared with the HLB group, the abundance of BSH-producing bacteria (e.g., *Bifidobacterium* and *Enterococcus*) and pathways related to Notch signaling and Wnt signaling were reduced in the HLAB group. **Conclusions:** This study revealed that berberine’s lipid-lowering and intestine-protective effects are closely related to the intestinal microbiota, especially BSH-producing bacteria.

## 1. Introduction

High-lipid diets are commonly used in fish farming due to their protein-sparing and growth-promoting effects [1,2]. However, long-term high lipid intake can lead to oxidative stress, metabolic ailments, inflammation, endoplasmic reticulum stress, excessive hepatic lipid accumulation, and intestinal microbiota dysbiosis [3,4]. Among these issues, fatty liver disease and intestinal damage are commonly observed and strongly linked to the intestinal microbiota. Specifically, microbiota-modulated bile acid (BA) metabolism influences nutrient utilization, energy metabolism, and inflammation. Primary bile acids, cholic acid (CA), and chenodeoxycholic acid (CDCA) are synthesized in the liver from cholesterol. They are conjugated with taurine or glycine to form bile salts like taurocholic acid (TCA) and taurochenodeoxycholic acid (TCDCA), which are stored in the gallbladder and secreted into the intestine. Microbial bile salt metabolism begins with bile salt hydrolase (BSH), which deconjugates bile salts, producing unconjugated bile acids (CA and CDCA). Subsequently, specific microbes convert CDCA or CA into secondary bile acids, such as lithocholic acid (LCA) and deoxycholic acid (DCA), through dehydrogenation reactions [1,5]. Typically, primary bile acids strongly activate the farnesoid X receptor (FXR), while many secondary bile acids, including glycine-β-muricholic acid (Gly-β-MCA) and ursodeoxycholic acid (UDCA), act as FXR antagonists. Activation of the hepatic FXR–small heterodimer partner (SHP) axis can reduce lipogenesis by inhibiting the expression of sterol regulatory binding element-protein 1c (SREBP1c) and its downstream genes (e.g., stearoyl-CoA desaturase 1 (SCD1) and fatty acid synthase (FAS)), and can also promote lipolysis by upregulating proliferator-activated receptor (PPAR) α and its target genes [1,6,7]. In addition, activation of intestinal FXR can increase ceramide production, thereby increasing energy metabolism and insulin sensitivity [5,8]. In particular, the Takeda G protein-coupled receptor 5 (TGR5)/cAMP signaling pathway is also influenced by bile acids, with the activation order LCA > DCA > CDCA > UDCA > CA [1,9,10]. Activation of TGR5 and FXR has anti-inflammatory properties, inhibiting the generation of inflammatory cytokines via the nuclear factor κB (NF-κB) pathway. This process safeguards intestinal barrier integrity, offering protection against colitis [11]. Thus, the deconjugation of bile salts by bacterial BSH stands out as a pivotal step, and increasing BSH activity can increase the proportion of unconjugated bile acids, thus activating the FXR and/or TGR5 pathways and reducing hepatic lipid accumulation and intestinal damage [1]. For example, *Bacillus cereus* is a BSH-producing bacterium, and feeding Nile tilapia (*Oreochromis niloticus*) a *Bacillus cereus*-supplemented high-carbohydrate diet improved intestinal and hepatic unconjugated and conjugated BAs, thus activating the hepatic FXR pathway and inhibiting lipogenesis [12]. In contrast, a high-lipid diet suppressed intestinal BSH activity, leading to diminished conversion of unconjugated primary bile acids (CDCA and CA) and subsequent inhibition of FXR pathway activation, causing fatty liver disease in grouper (*Epinephelus fuscoguttatus*♀ × *E. lanceolatus*♂) [13]. Therefore, supplementing with BSH-producing bacteria or incorporating feed additives to boost their population could effectively mitigate hepatic lipid accumulation and intestinal damage resulting from a high-lipid diet.

Berberine, an isoquinoline alkaloid (C_20_H_18_NO^+^), is prevalent in various herbal plants, including *Coptis chinensis* and *Berberis vulgaris*. With a longstanding historical usage in ancient Chinese medicine and Ayurvedic traditions, it has been used for alleviating conditions such as diarrhea, fatty liver, hypertension, and type 2 diabetes [14,15]. Recently, some studies have shown that berberine has promising effects on improving growth performance and immune and antioxidative status [16,17], reducing hepatic lipid accumulation [18,19], and maintaining liver and intestinal health in fish [17], thereby positioning it as a potential feed additive for protecting fish health. Nevertheless, berberine exhibits remarkably low oral bioavailability, typically less than 1%, with most ingested berberine remaining in the intestine. Its broad beneficial effects are primarily attributed to its ability to modulate the intestinal microbiota [20]. Notably, BSH-producing bacteria might be the target microbiota of berberine, but the modulation pattern is inconsistent in mammals [21,22,23]. Previously, we observed that berberine elevated the intestinal CA content and activated the intestinal and hepatic FXR pathways in yellow catfish (*Pelteobagrus fulvidraco*), leading us to hypothesize that this mechanism was strongly related to intestinal BSH-producing bacteria [16].

The zebrafish (*Danio rerio*) has become a valuable model organism for studying fish nutrition, intestinal microbiota, and lipid metabolism, primarily due to its genetic similarity to other fish species, ease of dietary manipulation, well-characterized metabolic pathways, susceptibility to metabolic disorders, and identification of core microorganisms [15,24]. In this study, we utilized an antibiotic cocktail to deplete the intestinal microbiota. We hypothesized berberine reduces hepatic lipids and improves intestinal health in a high-lipid diet by increasing BSH-producing bacteria and that antibiotics might diminish these effects by depleting microbiota.

## 2. Materials and Methods

All the experimental procedures adhered to the Guidelines for the Care and Use of Laboratory Animals in China. The Committee on the Ethics of Animal Experiments at Anhui Normal University approved the study (AHNU-ET2024038).

### 2.1. Diet Preparation

Table 1 presents the feed formulation and approximate compositions. The control diet consisted of 42% crude protein and 8% crude lipid. The HL diet had the same protein level, but had an increased lipid content (13%) due to the addition of corn oil and fish oil in a 1:1 ratio. The protein and lipid levels were designed according to previous publications [24,25]. Antibiotic cocktails (200 mg/kg neomycin, 200 mg/kg ampicillin, 200 mg/kg metronidazole, 200 mg/kg gentamicin, and 10 mg/kg vancomycin) [26] and 400 mg/kg berberine [16] were added to the HL diet alone and together. They were designated HLA, HLB, and HLAB, respectively. All ingredients were finely ground and passed through a 178 μm sieve. The powdered ingredients were then blended using a mixer, and water was added to enhance viscosity. The mixture was subsequently extruded through a 0.25 mm die using a feed extruder (model HKJ-218, Huarui, Wuxi, China). After extrusion, the diets were spread on a clean surface and air-dried with the assistance of an air conditioner and dehumidifier. Then, the pellets were pulverized into smaller particles, and particles with diameters between 0.63 and 0.85 mm were retained. A representative sample of the feed was collected for approximate composition analysis, while the remaining portion was sealed in plastic bags and stored at −20 °C until needed.

### 2.2. Feeding Trial

All wild-type zebrafish were purchased from Shandong YiXiYue Biotechnology Co., Ltd. (Weifang, China). After a two-week domestication, 300 fish (initial weight 0.10 ± 0.02 g) were distributed into 15 tanks (15 L, 20 fish per tank) under a circulating water system. The experimental fish were fed their corresponding diets daily at 8:00, 12:00, and 16:00 until apparent saturation for 30 days. The leftover food was collected one hour after the feeding via a siphon tube and then dried and weighed to calculate the consumption of each tank. During the feeding trial, the dead fish were monitored and removed every day, and tanks were cleaned as needed. The water condition was maintained at a temperature of 25.0 ± 0.5 °C, dissolved oxygen 6.40 ± 0.30 mg/L, ammonia 0.05 ± 0.01 mg/L, and pH 7.80 ± 0.05, as measured by the YSI (Yellow Springs, OH, USA).

### 2.3. Sampling

Four hours after the final feeding, intestinal contents from six fish per group were sampled, pooled, and transferred into germ-free tubes for 16S rDNA analysis. The remaining fish were subjected to a 20 h fasting period before being anesthetized with MS-222 (60 mg/L). Growth and morphological indices of each fish were recorded before dissection. Intestinal and liver samples were carefully preserved in 4% paraformaldehyde and liquid nitrogen for further analysis.

### 2.4. Enzyme, Biochemistry, and Histological Analysis

Hepatic triglyceride (TG) and total cholesterol (TC) levels were measured using diagnostic reagent kits from Nanjing Jiancheng Bioengineering Institute in Nanjing, China (TC, A110–1–1; TG, A110–1–1) according to the manufacturer’s protocols by spectrophotometry at 500 nm. The intestinal BSH activity was measured with a zebrafish BSH ELISA kit (XY-D6937) purchased from Shanghai Xuanya Biotechnology Co., Ltd., in Shanghai, China, and determined by spectrophotometry at 450 nm. The intestine samples were dehydrated (70%, 80%, 90%, 95%, and 100% alcohol gradually, followed by 100% xylene) and embedded in paraffin before being stained with hematoxylin and eosin (HE). Images of the intestines were acquired using an Olympus BX61 microscope from Tokyo, Japan. Histological parameters were subsequently measured using ImageJ 1.8.0 software.

### 2.5. Quantitative RT-PCR

Quantitative RT-PCR (qRT-PCR) analysis was performed by isolating total RNA from both the intestine and liver utilizing a MiniBEST universal RNA extraction kit from Takara, Japan. The liver and intestine tissues were homogenized beforehand by a homogenizer (Bkmam Biotechnology Co., Ltd., Changsha, China). An appropriate amount of lysis buffer was selected for homogenization based on the tissue size of the liver and intestine. Following RNA extraction, complementary DNA (cDNA) was synthesized using an Evo M-MLV RT Mix Kit with gDNA Clean for qPCR version 2, sourced from Accurate Biotechnology in Changde, China. The primer sequences for the targeted genes, including acetyl-CoA carboxylase (*accα*), cholesterol 7-alpha-monooxygenase (*cyp7a1*), carnitine palmitoyltransferase 1 (*cpt1*), fatty acid synthesis (*fas*), farnesoid X receptor (*fxr*), fibroblast growth factor 19 (*fgf19*), hormone-sensitive lipase (*hsl*), interleukin 1 beta (*il1β*), liver X receptor (*lxr*), nuclear factor κB (*nfκb*), peroxisome proliferator-activated receptor alpha (*pparα*), small heterodimer partner (*shp*), sterol regulatory element-binding protein 1c (*srebp1c*), Takeda G protein-coupled receptor 5 (*tgr5*), tumor necrosis factor α (*tnfα*), and zonula occludens 1 (*zo1*), were designed by Primer Premier 5.0, as detailed in Table 2. Analysis and detection were conducted using an SYBR Green Premix Pro Taq HS qPCR kit from Accurate Biotechnology in Hunan, China. The reaction process and calculation method were the same as those in our previous publication [16].

### 2.6. DNA Extraction and 16S rDNA Gene Sequencing

DNA extraction from the intestinal microbiota was carried out using an E.Z.N.A.^®^ Stool DNA Kit (D4015, Omega, Inc., Norcross, GA, USA). The concentration and purity of the extracted DNA were evaluated using a spectrophotometer (NanoDrop 8000, Thermo Fisher Scientific, Waltham, WI, USA). For amplification of the 16S rDNA region, domain-specific primers targeting the V4 region were employed: 806R (5′-GGACTACHVGGGTWTCTAAT-3′) and V4:515F (5′-GTGYCAGCMGCCGCGGTAA-3′). The PCR protocol followed Wang et al. [16]. Following PCR amplification, the amplicons were purified, and sequencing was performed using the Illumina NovaSeq platform at LC-Bio Technology Co., Ltd. in Hangzhou, China. The raw sequencing data were deposited in the NCBI Sequence Read Archive (SRA) database (PRJNA1092848).

### 2.7. Bioinformatic Analysis

Following the assignment of paired-end reads to samples via unique barcodes and the subsequent removal of barcode and primer sequences, the paired-end reads were merged using FLASH (version 1.2.7). Quality filtering of the raw reads was performed with fqtrim (v0.94) to obtain clean tags, ensuring high-quality data. Chimeric sequences were removed using Vsearch software (v2.3.4). Feature tables and sequences were generated through dereplication with DADA2. Alpha and beta diversities were then assessed by normalizing the sequences to a consistent count. Feature abundance was normalized by assessing the relative abundance of each sample using the SILVA classifier. Species diversity analysis included computations of the Shannon, observed species, and Simpson indices using QIIME2. Beta diversity calculations were conducted in QIIME2, with visualizations produced using the R package. Sequence alignment was carried out using BLAST, and annotations of the feature sequences were conducted with the SILVA database. Linear discriminant analysis effect size (LEfSe) analysis was employed to detect significant associations between bacterial taxa and distinct groups. Heatmap analysis was performed using heatmap version 1.0.12, utilizing the absolute abundance matrix of the microbiota. The predicted functional alterations in the microbiota across groups were assessed through PICRUSt2 analysis. Relative abundance comparisons among the various groups were evaluated using Welch’s *t*-test and multiple-test correction methods.

### 2.8. Data Analysis

The results are presented as means ± standard error (SE, n = 3). Normality and variance homogeneity were evaluated using the Kolmogorov–Smirnov and Levene’s tests, respectively. Statistical analysis was performed using one-way analysis of variance (ANOVA), followed by Duncan’s multiple range tests in IBM SPSS Statistics 20.0 to identify differences between treatments. Bacterial diversity and operational taxonomic unit (OTU) richness in the intestines of different fish groups were compared using the Mann–Whitney U test. *p* < 0.05 was considered to indicate statistical significance.

## 3. Results

### 3.1. Growth Performance, Morphological Parameters, and Feed Intake

As shown in Table 3, the HL group demonstrated a significantly lower survival rate compared to the Con group (*p* < 0.05). No significant differences were observed in the specific growth rates among the various groups (*p* > 0.05). The condition factor of the HLAB group was significantly lower than that of the HLB group, which was in turn significantly lower than that of the remaining three groups. Additionally, the feed intake in the HLB group was significantly higher than in the other groups.

### 3.2. Intestinal BSH Activity and Hepatic TC and TG Contents

**Figure 1:**
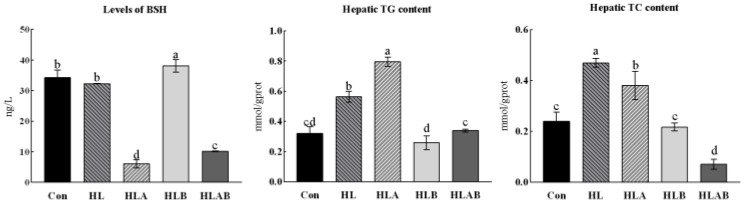
Intestinal bile-salt hydrolase (BSH) activity, hepatic total cholesterol (TC, mmol/gprot), and triglyceride contents (TG, mmol/gprot) of zebrafish fed different diets. Values are presented as means ± SE (n = 3). Mean values with different letters differed significantly (*p* < 0.05). Con, control group; HL, high-lipid group; HLA, antibiotic-supplemented high-lipid group; HLB, berberine-supplemented high-lipid group; HLAB, berberine- and antibiotic-supplemented high-lipid group.

In Figure 1, the BSH activity in the HL group was significantly higher than that in the HLA and HLAB groups, but significantly lower than that in the HLB group. Additionally, the HL group exhibited significantly higher levels of TC and TG compared to the Con group. In contrast, the HLA, HLB, and HLAB groups showed significantly reduced TC levels compared to the HL group. Moreover, while the TG level in the HLAB group significantly decreased compared to the HLA group, it was significantly higher than that in the HLB group.

### 3.3. Related Gene Expression

**Figure 2:**
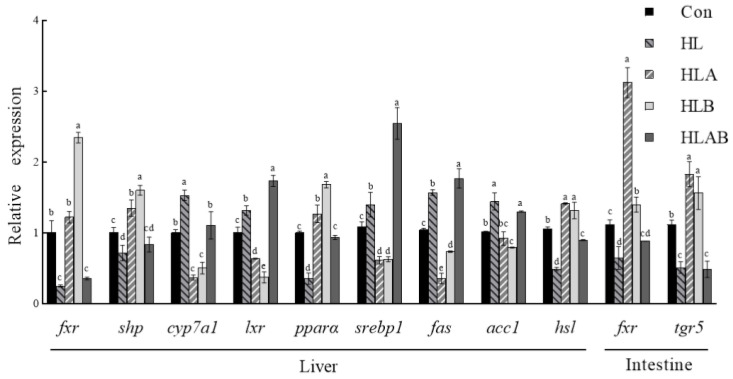
Relative gene expression levels of different groups fed the experimental diets. Acetyl-CoA carboxylase (*accα*), carnitine palmitoyltransferase1 (*cpt1*), cholesterol 7-alpha-monooxygenase (*cyp7a1*), fatty acid synthesis (*fas*), fibroblast growth factor 19 (*fgf19*), farnesoid X receptor (*fxr*), hormone-sensitive lipase (*hsl*), liver X receptor (*lxr*), peroxisome proliferator-activated receptor alpha (*pparα*), small heterodimer partner (*shp*), sterol regulatory element-binding protein 1c (*srebp1c*), and Takeda G protein-coupled receptor 5 (*tgr5*). Values are presented as means ± SE (n = 3). Mean values with different letters are significantly different (*p* < 0.05). Con, control group; HL, high-lipid group; HLA, antibiotic-supplemented high-lipid group; HLB, berberine-supplemented high-lipid group; HLAB, berberine- and antibiotic-supplemented high-lipid group.

In the liver, the HL group showed significantly downregulated *fxr*, *shp*, *pparα*, and *hsl* expression upregulatd expression of *cyp7a1*, *srebp1*, and *acc1* compared to the Con group (Figure 2). However, the *fxr*, *shp*, *pparα*, and *hsl* expression levels in the HLA and HLB groups were significantly higher than those in the HL and HLAB groups, following an opposite trend to the expression of *cyp7a1*, *lxr*, *srebp1*, *fas*, and *acc1*. Additionally, intestinal *tgr5* and *fxr* expression levels were significantly lower in the HL group compared to the Con group, whereas *tgr5* and *fxr* expression levels were significantly higher in the HLA and HLB groups than in the HL and HLAB groups.

**Figure 3:**
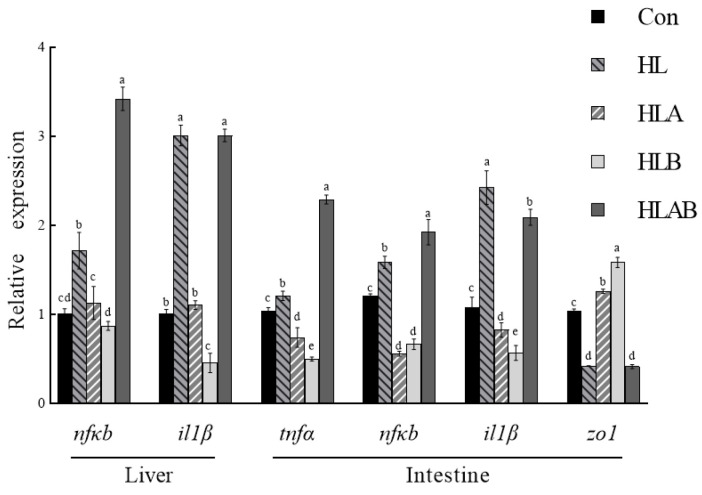
Inflammation and tight junction-related gene expression levels of different groups fed the experimental diets. Interleukin 1 beta (*il1β*), nuclear factor κB (*nfκb*), tumor necrosis factor α (*tnfα*), and zonula occludens 1 (*zo1*). Values are presented as means ± SE (n = 3). Mean values with different letters differed significantly (*p* < 0.05). Con, control group; HL, high-lipid group; HLA, antibiotic-supplemented high-lipid group; HLB, berberine-supplemented high-lipid group; HLAB, berberine- and antibiotic-supplemented high-lipid group.

The expression of proinflammatory cytokine genes, including *nfκb* and *il1β* in the liver, as well as *tnfα*, *nfκb*, and *il1β* in the intestine (Figure 3), were significantly elevated in the HL group compared to the Con group. However, hepatic nfκb and *il1β*, as well as intestinal *tnfα*, *nfκb*, and *il1β*, were significantly downregulated in the HLA and HLB groups, but were upregulated in the HLAB group compared to the HL group. Moreover, the intestinal expression level of *zo1* in the HL group was similar to that in the HLAB group, but significantly lower than that observed in the Con, HLA, and HLB groups.

### 3.4. Intestinal Histological Parameters

**Figure 4:**
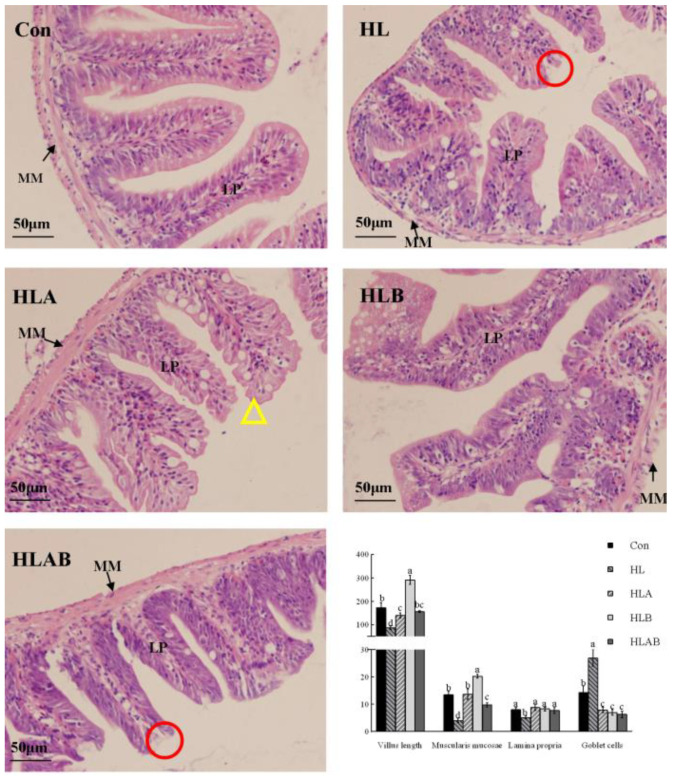
Intestinal structure and histological analysis of different groups. Circles represent intestinal mucous membrane shedding. Triangles represent the sawtooth of the villus edge. Values are presented as means ± SE (n = 3). Mean values with different letters differed significantly (*p* < 0.05). Con, control group; HL, high-lipid group; HLA, antibiotic-supplemented high-lipid group; HLB, berberine-supplemented high-lipid group; HLAB, berberine- and antibiotic-supplemented high-lipid group.

The HL group exhibited significantly lower villus length, lamina propria width, and muscularis mucosae width compared to the other groups. In contrast, the number of goblet cells followed an opposite trend among the various groups. Moreover, the villus structure of the HLA group appeared irregular, characterized by numerous serrations, while the remaining groups exhibited a regular, smooth structure (Figure 4).

### 3.5. Alpha and Beta Diversities of the Intestinal Microbiota

**Figure 5:**
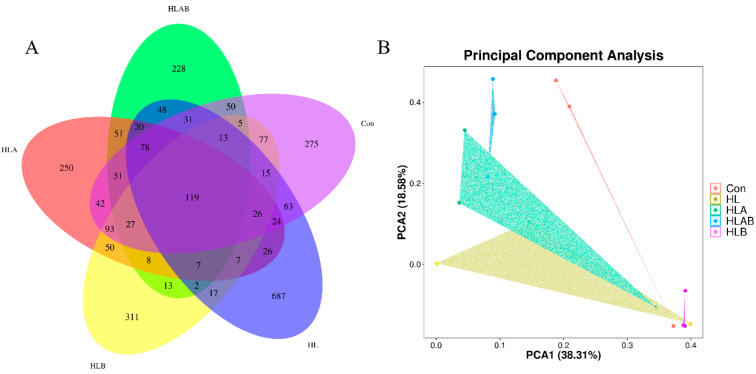
Venn diagram of the OTUs of intestinal microbiota under different treatments (n = 3) (**A**). PCA is based on the Bray–Curtis dissimilarity of zebrafish intestinal microbiota in different treatments (n = 3) (**B**). Con, control group; HL, high-lipid group; HLA, antibiotic-supplemented high-lipid group; HLB, berberine-supplemented high-lipid group; HLAB, berberine- and antibiotic-supplemented high-lipid group.

As depicted in Table 4, there were no significant differences in the number of observed OTUs, Shannon index, Simpson index, or Pielou-E across the various treatments. Collectively, the five groups shared 119 operational taxonomic units (OTUs), with the HL group displaying the highest number of OTUs (687), followed by the HLB (311), Con (275), HLA (250), and HLAB (228) groups (Figure 5A). Principal component analysis (PCA) indicated that the first two principal components (PCA1 and PCA2) explained 38.31% and 18.58% of the total variance, respectively (Figure 5B).

### 3.6. Taxonomic Composition Analysis

**Figure 6:**
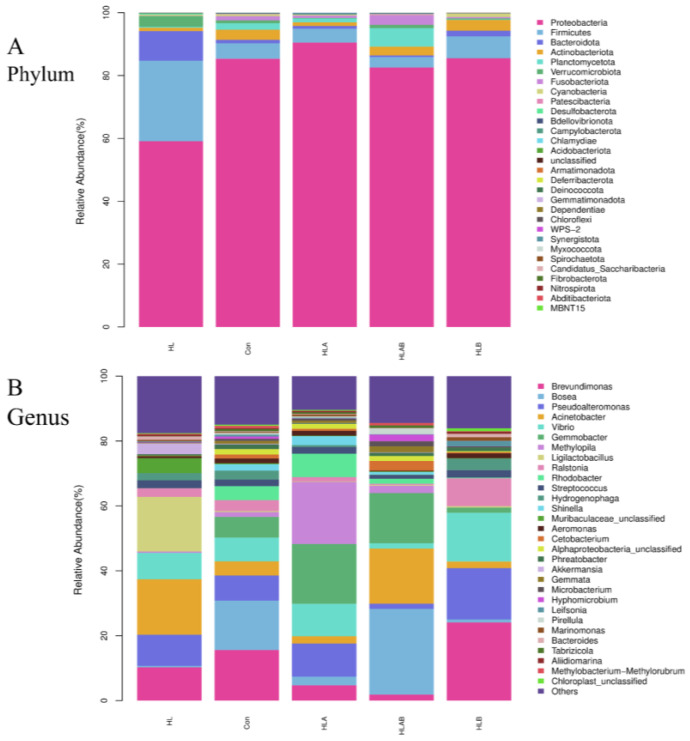
Taxonomy classification of reads at phylum (**A**) and genus (**B**) levels along with Bray–Curtis distance-based clustering analysis (n = 3). The top 30 enriched bacterial phyla and genera are shown in this figure; “Others” represents the unshown phyla and genera. Con, control group; HL, high-lipid group; HLA, antibiotic-supplemented high-lipid group; HLB, berberine-supplemented high-lipid group; HLAB, berberine- and antibiotic-supplemented high-lipid group.

Proteobacteria was the dominant phylum across all groups, followed by Firmicutes, Bacteroidota, Actinobacteriota, and Planctomycetota in terms of relative abundance. Notably, the HL group exhibited a higher prevalence of Firmicutes compared to the other groups (Figure 6A and Table S1). The HL and HLB groups demonstrated significantly lower abundance of Planctomycetota and Patescibacteria compared to the HLAB group (Table S1). The dominant genera in the different treatment groups were Brevundimonas, Bosea, Pseudoalteromonas, Acinetobacter, and Vibrio. The abundance of Brevundimonas was significantly higher in the HLB group than in the HLAB group (Figure 6B and Table S1).

**Figure 7:**
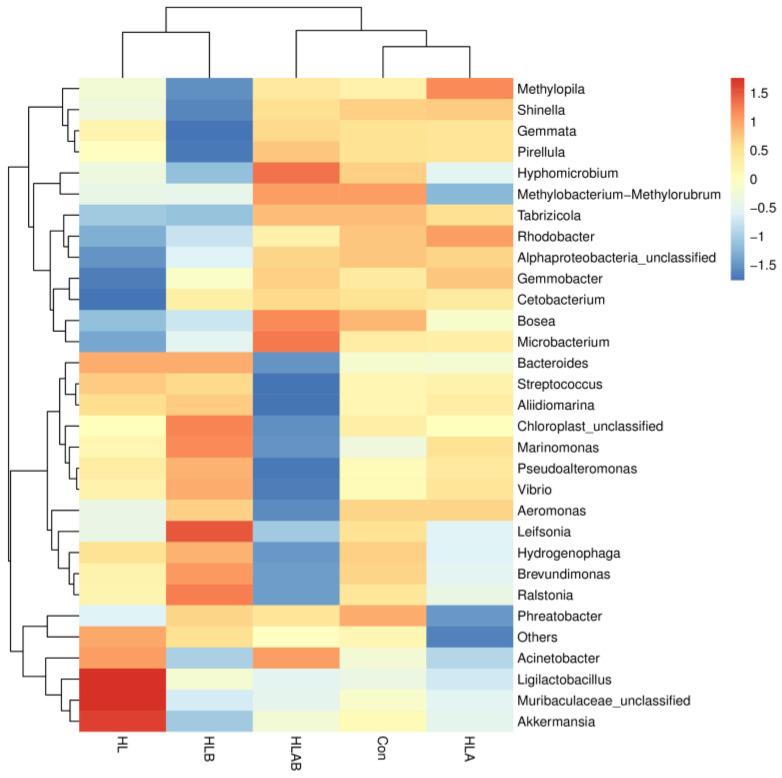
Heat map of top 30 genera of zebrafish intestinal microbiota under different treatments. Con, control group; HL, high-lipid group; HLA, antibiotic-supplemented high-lipid group; HLB, berberine-supplemented high-lipid group; HLAB, berberine- and antibiotic-supplemented high-lipid group.

Figure 7 illustrates a heatmap depicting the top 30 genera. In comparison to the Con group, the abundance of *Ligilactobacillus*, *Muribaculaceae-unclassified*, and *Akkermansia* was higher, whereas the abundance of *Bosea*, *Microbacterium*, *Cetobacterium*, *Gemmobacter*, *Alphaproteobacteria*-unclassified, *Rhodobacter*, and *Tabrizicola* was lower in the HL group. Compared to the HL group, the abundance of *Ralstonia*, *Leifsonia*, *Brevundimonas*, and *Marinomonas* in the HLB group increased, but the abundance of *Akkermansia*, *Methylopia*, *Shinella*, *Gennata*, and *Pirellula* decreased. The HLAB group had lower abundance of *Bacteroides*, *Streptococcus*, *Aliidiomarina*, *Marinomonas*, *Pseudoalteromonas*, *Vibrio*, *Aeromonas*, *Leifsonia*, *Hydrogenophaga*, *Brevundimonas*, and *Ralstonia*, but higher abundance of Bosea, Microbacterium, and Hyphomicrobium than the HLB group.

**Figure 8:**
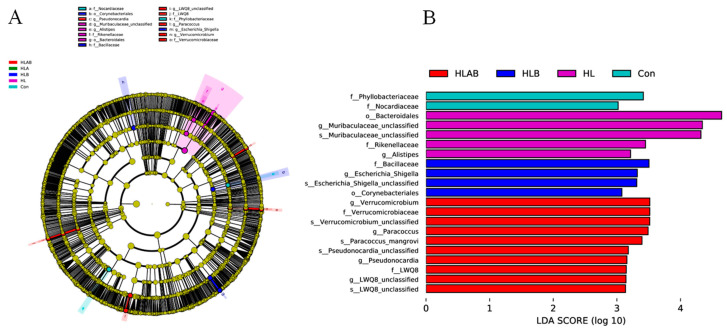
Cladogram showing the phylogenetic distribution of the bacterial lineages associated with different treatments. Taxonomic representation of statistically and biologically consistent differences between intestinal microbiota of zebrafish under different treatments (**A**). Histogram of linear discriminant analysis (LDA) scores for differentially abundant taxa (**B**). Con, control group; HL, high-lipid group; HLA, antibiotic-supplemented high-lipid group; HLB, berberine-supplemented high-lipid group; HLAB, berberine- and antibiotic-supplemented high-lipid group.

The cladogram illustrates taxa exhibiting significant differences between groups at both the phylum and genus levels (Figure 8A). LEfSe analysis serves as an analytical tool for identifying and interpreting biometric markers within high-dimensional datasets. The LDA distribution histogram indicates that all biomarkers had LDA scores exceeding 3.0. The biomarkers in the Con group were *Phyllobacteriaceae* and *Nocardiaceae*. *Bacteroidales*, *Muribaculaceae*-unclassified, *Rikenellaceae*, and *Alistipes* were biomarkers for the HL group. *Bacillaceae*, *Escherichia Shigellas*, *Escherichia Shigella*-unclassified, and *Corynebacteriales* were the biomarkers of the HLB group. *Verrucomicrobiaceae*, *Verrucomicrobium*, *Paracoccus*, *Paracoccus mangrove*, *Verrucomicrobium*-unclassified, *Pseudonocardia*, *Pseudonocardia* unclassified, LWQ8, and LWQ8-unclassified were the biomarkers of the HLAB group.

Spearman correlation analysis (Figure S1) revealed that antibiotic intervention exhibited a significant positive correlation with the abundance of *Bosea*, *Microbacterium*, *Gemmobacter*, *Pirellula*, *Methylopila*, *Gemmata*, *Tabrizicola*, *Shinella*, *Alphaproteobacteria*-unclassified, and *Rhodobacter*, but was significantly negatively related to the abundance of *Alilildiomarina*, *Streptococcus*, *Hydrogenophaga*, *Brevundimonas*, *Ralstonia*, *Leifsonia*, *Marinomonas*, *Bacteroides*, *Pseudoalteromonas*, and *Vibrio*. Additionally, berberine was significantly negatively related to *Muribaculaceae*-unclassified abundance.

### 3.7. Functional Prediction Analysis of Intestinal Microbiota

**Figure 9:**
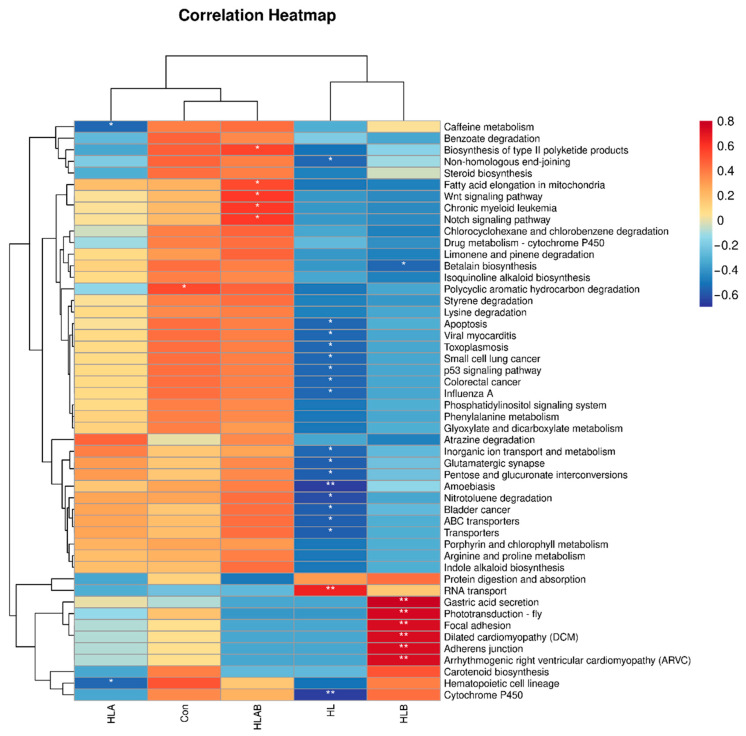
Function prediction analysis of gut microbiota by Spearman correlation analysis. Differences in the relative abundance of level 3 KEGG pathways. Con, control group; HL, high-lipid group; HLA, antibiotic-supplemented high-lipid group; HLB, berberine-supplemented high-lipid group; HLAB, berberine- and antibiotic-supplemented high-lipid group. * *p* < 0.05, ** *p* < 0.01.

PICRUSt2 and Spearman correlation analysis were utilized to predict and evaluate microbiota-associated KEGG pathways (Figure 9 and Figure S2). At KEGG level 1, the HL group showed lower enrichment in multiple pathways, while the HLAB group was more enriched. At level 2, the HL group had lower enrichment in most pathways, except immune-related ones. At level 3, the HL group showed lower abundance in various pathways but higher in “RNA transport,” while the HLB group showed higher abundance in several pathways, including gastric acid secretion and cardiomyopathy-related pathways, and the HLAB group showed higher enrichment in biosynthesis and signaling pathways.

## 4. Discussion

In the present study, a high-lipid diet resulted in significantly higher mortality in zebrafish compared to the control diet, consistent with previous findings in blunt snout bream (*Megalobrama amblycephala*) [27] and grass carp (*Ctenopharyngodon idella*) [28]. However, the HLA group exhibited the lowest weight gain, indicating that antibiotic treatment hindered growth. Similarly, supplementation with 2.00 g/kg oxytetracycline (OTC) in a high-lipid diet significantly reduced weight gain, body protein content, and feed efficiency in Nile tilapia [29]. This could be due to antibiotic treatment increasing energy expenditure [30], excessive reactive oxygen species generation [31], and impaired intestinal health [30]. However, berberine supplementation in the high-lipid diet improved the growth performance of zebrafish. Enhanced weight gain by berberine has been reported in blunt snout bream [18,32], largemouth bass (*Micropterus salmoides*) [33,34], and Nile tilapia [35]. The growth-promoting effect of berberine was attributed to its strong antioxidative and anti-inflammatory properties, maintenance of intestinal microbiota homeostasis, and safeguarding of liver and intestinal health in fish [17], as well as the improvement in feed intake in the present study. Moreover, antibiotic intervention did not inhibit the growth-promoting effect of berberine, but berberine ameliorated the negative effect of antibiotics on growth performance. A similar result has been reported in largemouth bass fed 1 g/kg berberine or 0.9 g/kg antibiotic alone or both simultaneously [34].

The present study revealed that berberine supplementation significantly increased intestinal BSH activity, aligning with reports that berberine increases the abundance of the BSH-producing bacteria *Lactobacillus* and *Roseburia* in mice [23]. In contrast, the HLA and HLAB groups had significantly lower BSH activity than the other groups, which might be strongly related to antibiotically reduced abundance of total intestinal microbiota, including the BSH-producing bacteria. An analogous effect of antibiotic mixtures in inhibiting intestinal BSH activity has been observed in grass carp [26]. Normally, increased intestinal BSH activity results in improved unconjugated primary acid content, thus activating the FXR pathway; however, the *fxr* expression level in the HLA group was still upregulated. Previously, both antibiotic-mediated inhibition [26] and activation [36] of the FXR pathway have been reported in fish. We postulate that antibiotics may decrease the abundance of secondary bile acid-producing bacteria, consequently reducing FXR antagonist levels.

The present study demonstrated that a high-lipid diet led to excessive hepatic TG and TC accumulation, a hallmark of fatty liver disease in fish [3,37], and is linked to suppression of the hepatic FXR pathway. Previously, a high-lipid diet was found to decrease the activity of BSH and the levels of unconjugated primary bile acids, consequently impeding activation of the FXR and TGR5 pathways. This inhibition led to excessive accumulation of lipids in the liver of the grouper [13]. However, in the present study, the hepatic TC content was significantly reduced by antibiotics, berberine, and their combination. Accordingly, the hepatic lipogenesis genes *lxr*, *srebp1*, *fas*, and *acc1* were downregulated, whereas the lipolysis genes *pparα* and *hsl* were upregulated in the HLA and HLB groups, which was consistent with the activation of the FXR–SHP axis. However, the antibiotics attenuated the activation of *fxr* induced by berberine, possibly because the intestinal BSH activity in the HLAB group was significantly lower than that in the HLB group, as demonstrated by the significantly reduced abundance of *Bifidobacterium* and *Enterococcus* in the present study (Table S1), which was positively correlated with the BSH content [38]. Dietary berberine-induced activation of the hepatic and/or intestinal FXR pathways has been reported in yellow catfish [16] and largemouth bass [33]. Additionally, dietary berberine has been reported to promote hepatic lipolysis and/or inhibit lipogenesis in black sea bream (*Acanthopagrus schlegelii*) [2], yellow drum (*Nibea albiflora*) [39], and blunt snout bream [18,40]. Studies in zebrafish also revealed that berberine supplementation in a high-cholesterol diet significantly increased lipid metabolism, reduced serum TC and TG contents, and reduced hepatic lipid accumulation [15,41]. Interestingly, antibiotic supplementation in the high-lipid diet significantly reduced the hepatic TC content, but significantly increased the TG content, which indicates that TG and TC have distinct responses to antibiotic intervention. Inconsistent effects of antibiotics on lipid metabolism have been reported across fish species. For example, antibiotic supplementation in a medium- or high-lipid diet significantly reduced hepatic lipid level, TC, and TG contents in hybrid grouper, mainly by upregulating lipolysis-related gene expression in the medium-lipid diet group while downregulating lipogenesis-associated gene expression in the high-lipid diet group [42]. Similarly, prolonged exposure to waterborne OTC also reduced lipid accumulation and increased energy consumption in zebrafish [43]. However, an antibiotic cocktail caused significantly higher TG and TC contents in the liver of grass carp [26], and a broad-spectrum antibiotic reduced hepatic steatosis in zebrafish [36]. Xu et al. [42] concluded that the differences in hepatic TC and TG accumulation caused by antibiotic intervention are related to the form, dose, type, period, and usage of antibiotic, as well as species and diet component differences.

Normally, a high-lipid diet activates NF-κB-mediated inflammation [4], which was also proven in this study. However, antibiotics or berberine significantly downregulated the overexpression of the proinflammatory genes *nfκb* and *il1β* in the liver and the proinflammatory genes *nfκb*, *il1β*, and *tnfα* in the intestine caused by the high-lipid diet. This effect is likely attributed to the upregulation of FXR and TGR5 pathways in the liver and/or intestine, as activation of these pathways can directly suppress NF-κB-dependent proinflammatory cytokine induction, including IL1β and TNFα [8,11]. Previously, berberine downregulated proinflammatory cytokine expression in many fish species [19,39,44]. In addition, broad-spectrum antibiotics that activate the intestinal FXR pathway and reduce the expression of inflammatory genes (such as *il1β*, *tnfα*, *il6*, *il10*, and *infγ*) have also been reported in zebrafish [36]. Interestingly, the HLAB group had significantly higher expression levels of these proinflammatory genes in both the liver and intestine, which was strongly related to the inhibition of the FXR and TGR5 pathways in the HLAB group. In addition, reduced BSH activity in the HLAB group retarded the hydrolysis of conjugated bile acids into free bile acids, which are intolerant to many pathogenic bacteria [45]; thus, increased pathogenic bacterial richness (discussed below) could be another contributing factor to intestinal inflammation.

A high-lipid diet typically promotes increased mucin secretion in the intestines, which helps reduce inflammation and causes the host to produce more mucin to preserve intestinal barrier function This leads to the establishment of a viscoelastic protective layer composed of mucins secreted primarily by goblet cells [4]. In line with this, an increase in the number of goblet cells induced by a high-lipid diet was found in the present study, as well as studies on black sea bream [33] and zebrafish [4]. Antibiotic or berberine treatment increased villus length and reduced goblet cell numbers, indicating improved intestinal structure and health. Conversely, reduced villus length, muscularis mucosae thickness, and lamina propria are considered intestinal damage [46], and are commonly observed in high-lipid diet-fed fish [3,33,47]. In addition, a high-lipid diet caused intestinal mucous membrane shedding and villi to fall off in grass carp [28], which was also observed in the present study. Interestingly, the HLA group exhibited many sawteeth on the villus edge, indicating damage to the microvilli, which might be a side effect of antibiotics. Previous studies revealed that antibiotic treatment distorted intestinal histomorphology and tight junction proteins in fish [29,30]. On the other hand, berberine has been widely reported to improve intestinal structure in fish, mainly by reducing inflammation and inhibiting pathogenic bacteria while promoting probiotic colonization, especially that of short-chain fatty acid (SCFA)-producing bacteria [16,17]. Notably, the HLAB group exhibited obvious mucous membrane shedding and significantly reduced muscularis mucosae thickness in the intestine. This morphological change was proven by the significantly lower expression level of *zo1*, which is a tight junction protein-encoding gene of the intestine. Reduced levels of tight junction proteins in the intestines disrupt the integrity of the tight junction barrier, resulting in heightened intestinal permeability [28].

A balanced intestinal microbiota is essential for maintaining the host’s nutritional, physiological, and metabolic functions [42]. In the present study, different treatments did not significantly influence the alpha diversity of the intestinal microbiota. Many studies have reported that antibiotics reduce intestinal microbial richness in fish [30,48]. However, antibiotics or berberine did not significantly change the intestinal alpha diversity of largemouth bass [34], grass carp [26], yellow catfish [16], or black sea bream [33]. Notably, principal component analysis (PCA) results demonstrated that the microbial compositions of the different groups were distinctly separate.

Previously, high-lipid diets have been observed to induce intestinal damage, closely associated with microbiota dysbiosis, characterized by a reduction in probiotic bacteria and an increase in pathogenic bacterial growth in fish [33,43]. In the HL group, the abundance of Firmicutes, Bacteroidota, Nitrospirota, and Verrucomicrobiota increased, whereas that of Proteobacteria and Fusobacteriotain decreased. Accordingly, Verrucomicrobiota was negatively related to FXR activation in common carp (*Cyprinus carpio* L.) [49]. Similarly, a high-lipid diet caused a higher abundance of Bacteroidetes in zebrafish [4]. The findings of the current study suggest that the HL diet led to a decrease in the abundance of certain probiotics, including *Microbacterium* [50,51], *Cetobacterium* [52], and *Gemmobacter* [53]. Interestingly, the HL group also exhibited high abundance of some probiotics, especially *Ligilactobacillus* (belonging to *Lactobacillus*) and *Akkermansia*, possibly because the damaged intestine of the HL group was recovering. *Akkermansia* is a probiotic associated with anti-inflammatory properties and is involved in numerous metabolic processes [16], and *Lactobacillus* is a probiotic bacteria that reduces intestinal injury [54]. A previous study revealed that the abundance of both *Akkermansia* and *Lactobacillus* significantly increased in the chronic recovery phase of the disease, suggesting their potential role in reversing intestinal damage [55]. According to the KEGG level 3 analysis, the HL group showed significantly lower enrichment of the cytochrome P450 pathway. Hepatocytes synthesize primary bile acids through cholesterol oxidation by cytochrome P450 [56]; thus, a high-lipid diet might interfere with bile acid synthesis. Additionally, the p53 signaling pathway and ABC transporters were also significantly inhibited in the HL group. p53 is a well-known tumor suppressor protein that undergoes rapid activation and accumulates in the nucleus, where it controls the transcription of genes related to cell cycle arrest, DNA repair, and apoptosis and is involved in immune defense and antiviral responses [57,58]. The inhibition of ABC transporters by high-fat diets has been widely reported in various animals [59,60]. The ABC transporter can promote the efflux of cellular cholesterol to HDL and inhibit hepatic cholesterol accumulation [61], and microbiota-mediated inhibition of this transporter might be a contributing factor to hepatic lipid accumulation in the HL group.

In the present study, *Escherichia Shigella* was identified as a biomarker of the HLB group. Its abundance was increased in yellow catfish fed 400 mg/kg berberine and was positively related to the production of the FXR agonists CA and ethyl-chenodeoxycholic acid (OCA) [16], as well as in rats fed with a berberine-supplemented diet [62,63]. In particular, *Escherichia Shigella* is positively related to BSH production [38] and is able to produce butyrate [64], which might facilitate the hydrolysis of bile salts into primary BAs, activate the hepatic–intestinal FXR signaling pathway, or improve intestinal energy metabolism [16,63]. Moreover, the HLB group had a significantly higher abundance of *Enterococcus*, which is also a BSH-producing bacteria [38], than the HL group. The abundance of *Ralstonia* was significantly higher in the HLB group than in the other groups, which was also observed in berberine-fed yellow catfish and was positively related to the FXR agonists CA and OCA [16]. Bile salt supplementation also increased *Ralstonia* richness in gilthead seabream (*Sparus aurata*) [65]. At the KEGG 3 level, the HLB group demonstrated significantly higher enrichment in adherens junction and focal adhesion pathways, which are pivotal parts of the junction structure and are involved in epithelial cell turnover, epithelial integrity, and structural stability [66,67]. These findings were supported by the enhanced intestinal integrity and upregulated *zo1* expression in this group. In addition, the HLB group had a significantly greater abundance of gastric acid secretion. Gastric acid secretion plays a vital role in selecting potential probiotics in the gut [68] and constitutes an essential chemical barrier that protects intestinal mucosa from erosion by digestive enzymes and other chemical substances [69].

The regulation of intestinal microbiota by antibiotics is complex. Although the HLA group did not show enrichment of any biomarker bacteria, correlation analysis indicated that antibiotics were negatively associated with certain pathogenic bacteria, such as *Vibrio* [70] and *Streptococcus* [71], as well as positively related to the abundance of some pathogenic bacteria, such as *Rhodobacter* [72] and *Shinella* [73]. In addition, antibiotics were positively related to some probiotic bacteria, such as *Pirellula* [74] and *Gemmata* [75], but negatively related to some probiotics, such as *Pseudoalteromonas* [47,76], *Ralstonia* [65], *Brevundimonas* [33,77,78], *Leifsonia* [12], and *Hydrogenophaga* [33,79]. Specifically, *Bacteroides* spp. are SCFA-producing bacteria [80] and negatively related to antibiotics. Thus, the impaired intestinal morphological structure in the HLA and HLAB groups might be partially attributed to the fact that antibiotics reduce the intestinal SCFA content, which can stimulate protein-coupled receptors or traverse cell membranes, enhancing cell signaling and regulating the immune response. Furthermore, SCFAs could impact the turnover of enterocytes, particularly in conditions where the mucus layer is not in optimal condition [81]. Similarly, long-term ingestion of antibiotics increased the vulnerability of zebrafish to infections [30], and florfenicol-containing feed induced a high abundance of potentially pathogenic bacteria in the intestine of channel catfish (*Ictalurus punctatus*) [82].

Compared with that in the HLB group, the abundance of *Bifidobacterium*, *Enterococcus*, and *Brevundimonas* was significantly lower in the HLAB group (Table S1). *Brevundimonas* is rarely studied in fish, but is typically considered a beneficial bacterium. Its abundance was high in healthy fish [33,77,78] and those fed bile acids [65], but low in *Aeromonas hydrophila*-infected zebrafish [83]. *Brevundimonas* is reportedly involved in bile acid metabolism through its 7α-hydroxysteroid dehydrogenase (7α-HSD) activity, which is crucial for the formation of secondary bile acids [56]. Some potential pathogenic bacteria, such as *Paracoccus* and *Pseudonocardia*, were identified as biomarkers of the HLAB group. The abundance of *Paracoccus* increased in response to exposure to some heavy metals [84,85,86] or polystyrene nanoplastics [87] in fish. *Pseudonocardia* has also been identified as a pathogenic bacteria [16,88]. Moreover, the HLAB group was significantly enriched in the Wnt signaling pathway and Notch signaling pathway. The Wnt signaling pathway forms an intricate network of protein interactions that are primarily active during embryonic development and cancer and play a role in the regular physiological processes of adult organisms [89]. Specifically, intestinal epithelial cell proliferation in zebrafish is stimulated by activation of Wnt signaling [90]. In addition, abnormal differentiation of the intestinal epithelium and intestinal barrier dysfunction are associated with depression of the Notch signaling pathway [91]. The interaction between the Wnt and Notch signaling pathways regulates intestinal cell proliferation and mucus production, aiding in the restoration of barrier integrity [92].

## 5. Conclusions

Our findings demonstrated that berberine effectively counteracts the negative impact of high lipid intake, leading to reduced hepatic TC and TG levels, decreased lipogenesis, and suppressed inflammation, mainly by activating the FXR and TGR5 pathways. Additionally, we found that the benefits of berberine are closely associated with its influence on the intestinal microbiota. Fish receiving berberine exhibited increased levels of beneficial probiotic bacteria, particularly BSH-producing bacteria, while antibiotic treatment resulted in unfavorable changes in microbiota composition, reduced beneficial bacteria potentially promoted harmful pathways, and attenuated the beneficial effects of berberine. Overall, our findings emphasize the crucial role of the intestinal microbiota in berberine-mediated lipid-lowering and intestine-protective effects and highlight the risks associated with antibiotic use to fish health and productivity.

## Figures and Tables

**Table 1:** Feed formulation (air-dried basis) and approximate compositions of the experimental diets ^1^.

Ingredients (g/kg)	Con	HL	HLA	HLB	HLAB
Fishmeal	300	300	300	300	300
Fermented soybean meal	250	250	250	250	250
Corn meal	180	180	180	180	180
Corn gluten meal	80	80	80	80	80
Corn oil	10	30	30	30	30
Fish oil	10	30	30	30	30
Soybean lecithin	20	20	20	20	20
Vitamin premix ^2^	15	15	15	15	15
Mineral premix ^3^	15	15	15	15	15
Lysine	10	10	10	10	10
Methionine	10	10	10	10	10
Berberine ^4^	0	0	0	0.4	0.4
Antibiotic cocktail ^5^	0	0	2.43	0.00	2.43
Sodium alginate	15	15	15	15	15
Carboxymethylcellulose	59.5	19.5	19.1	18.29	18.69
CaHPO_4_·2H_2_O	20	20	20	20	20
Betaine	5	5	5	5	5
Antioxidants	0.5	0.5	0.5	0.5	0.5
Total	1000	1000	1000	1000	1000
Approximate analyzed composition					
Moisture	52.9	54.6	60.2	61.6	59.3
Crude protein	422.3	415.5	416.8	411.1	420.1
Crude lipid	81.7	128.0	130.5	126.7	127.3
Ash	96.4	90.5	92.3	92.1	93.5

^1^ Con, control group; HL, high-lipid group; HLA, antibiotic-supplemented high-lipid group; HLB, berberine-supplemented high-lipid group; HLAB, berberine- and antibiotic-supplemented high-lipid group. ^2^ Vitamin premix (mg/kg of diet): retinyl acetate, 40; cholecalciferol, 0.1; DL-α-tocopheryl acetate, 80; menadione, 15; niacin, 165; riboflavin, 22; pyridoxine HCl, 20; thiamin mononitrate, 45; D-Ca pantothenate, 102; folic acid, 10; vitamin B-12, 0.9; inositol, 450; ascorbic acid, 150; Na menadione bisulfate, 5; thiamin, 5; choline chloride, 320 and aminobenzoic acid, 50. ^3^ Mineral premix (mg/kg of diet): Na_2_SiO_3_, 0.4; CaCO_3_, 350; NaH_2_PO_4_·H_2_O, 200; KH_2_PO_4_, 200; MgSO_4_·7H_2_O, 10; MnSO_4_·H_2_O, 2; CuCl_2_·2H_2_O, 1; ZnSO_4_.7H_2_O, 2; FeSO_4_·7H2O, 2; NaCl, 12; KI, 0.1; CoCl_2_·6H_2_O, 0.1; Na_2_MoO_4_·2H_2_O, 0.5; AlCl_3_·6H_2_O, 1; and KF, 1. ^4^ Berberine (HPLC ≥ 98%) was purchased from the Spring and Autumn biotechnology company, in Nanjing, China. ^5^ Antibiotic cocktail: 200 mg/kg ampicillin, 200 mg/kg neomycin, 200 mg/kg gentamicin, 200 mg/kg metronidazole, and 10 mg/kg vancomycin [26].

**Table 2:** The primers for qRT-PCR.

Gene	Primer Sequences (5′to·3′)	Accession Number
*accα*	F: AGGAGGACAGCAAGAGCATT	NM_001271308.1
	R: TGATCTGTCGGTCTTTGTGC	
*cpt1*	F: GTCCCGATCAGTAGGTACA	NM_001044854.1
	R: TCCCATTGAGCAGAACAGAG	
*cyp7a1*	F: ACCTTCAACGAGCTGAGCAA	NM_201173.2
	R: TGTCCAACTGCTCCCTTGTC	
*fas*	F: CAGATAAAGTGCAGACTGAGGAAGC	XM_685355.7
	R: GTATGACCTACAGTACGACTGCTCA	
*fgf19*	F: AGCTCGGACAGTAAGTTTGAT	NM_001012246.2
	R: TTGTAGCCGTCTGGAAGGATG	
*fxr*	F: ACATCGTGCATGATCCGTC	XM_005166733.4
	R: GCACTTCTGTAAGCAGACACTC	
*hsl*	F: GCAATCCATCTACGTTGGTACT	XM_005159495.4
	R: CGTCTCATATGCATTGCCAGT	
*il1β*	F: CTGGAGATGTGGACTTCGCA	NM_212844.2
	R: CGTTCACTTCACGCTCTTGG	
*lxr*	F: TCTTCTCAGCAGACCGACCA	YP_006568.1
	R: CGTAGGCTGACCAGCTTCAT	
*nfκb*	F: CTAACTACAGCGGACACACG	NM_213184.2
	R: CAGGTCTACGGCCAAATGGA	
*pparα*	F: CTGCGGGACATCTCTCAGTC	NM_001102567.1
	R: CTCGACATCTCGTTCTCCCG	
*shp*	F: TTAGCGACATCTCGCCACTC	NM_001256191.1
	R: CCATTGCACTTGCACCTTCC	
*srebp1*	F: CAGCCGCAGTTCATTAAGGC	NM_001105129.1
	R: ACGTCCACTTCCATGGTCAC	
*tgr5*	F: CTGGAGCGCCTGCTCTT	XM_017357898.2
	R: CAGCGAGTCCACGAGTATCC	
*tnfα*	F: AGCAGCATGGTGAGATACGA	NM_001024447.1
	R: CCTTCTTCGTTTGGCTTCATCA	
*zo1*	F: CCTCTCCCCTACCTCACACA	XM_009303250.3
	R: GTACCATGCCGCTAGGACC	
*β-actin*	F: GGACTCTGGTGATGGTGTGA	EU161066
	R: CTGTAGCCTCTCTCGGTCAG	

Abbreviation: *accα*, acetyl-CoA carboxylase; *cyp7a1*, cholesterol 7-alpha-monooxygenase; *cpt1*, carnitine palmitoyltransferase 1; *fas*, fatty acid synthesis; *fxr*, farnesoid X receptor; *fgf19*, fibroblast growth factor 19; *hsl*, hormone-sensitive lipase; *il1β*, interleukin 1 beta; *lxr*, liver X receptor; *nfκb*, nuclear factor κB; *pparα*, peroxisome proliferator-activated receptor alpha; *shp*, small heterodimer partner; *srebp1c*, sterol regulatory element-binding protein 1c; *tgr5*, Takeda G protein-coupled receptor 5; *tnfα*, tumor necrosis factor α; *zo1*, zonula occludens 1.

**Table 3:** Effects of different diets on growth performance, condition factor, and feed intake of zebrafish ^1^.

Index ^2^	Con	HL	HLA	HLB	HLAB
SR	91.11 ± 2.22 ^a^	73.33 ± 3.84 ^b^	82.22 ± 3.85 ^ab^	82.22 ± 3.85 ^ab^	84.44 ± 4.44 ^ab^
FW	0.30 ± 0.06 ^ab^	0.29 ± 0.06 ^ab^	0.27 ± 0.04 ^b^	0.47 ± 0.08 ^a^	0.45 ± 0.15 ^ab^
WG	206 ± 37 ^ab^	193 ± 35 ^ab^	173 ± 27 ^b^	370 ± 50 ^a^	350 ± 90 ^ab^
SGR	3.06 ± 0.83 ^abc^	3.22 ± 0.67 ^ab^	3.31 ± 0.34 ^a^	5.11 ± 0.37 ^c^	4.88 ± 0.63 ^bc^
CF	4.35 ± 0.13 ^a^	4.31 ± 0.19 ^a^	4.80 ± 0.09 ^a^	3.53 ± 0.30 ^b^	2.33 ± 0.21 ^c^
FI	9.30 ± 0.14 ^c^	11.00 ± 0.28 ^bc^	11.20 ± 1.09 ^bc^	16.28 ± 0.12 ^a^	12.07 ± 0.61 ^b^

^1^ Mean values ± standard error (SE, n = 3) are presented for each group; values with different superscripts in the same row differ significantly (*p* < 0.05). Con, control group; HL, high-lipid group; HLA, antibiotic-supplemented high-lipid group; HLB, berberine-supplemented high-lipid group; HLAB, berberine- and antibiotic-supplemented high-lipid group. ^2^ SR (survival rate, %) = 100 × (final fish number/initial fish number); FW (final weight, g); WG (weight gain, %) = 100 × (final body weight − initial body weight)/initial body weight; SGR (specific growth rate, % day^−1^) = 100 × (ln final body weight − ln initial body weight)/days; CF (condition factor, mg mm^−3^) = final body weight/final body length^3^ × 100; FI (feed intake, mg/fish/day) = dry diet feed/final fish number/days.

**Table 4:** Alpha diversity of different treatments ^1^.

Index	Con	HL	HLA	HLB	HLAB
Observed OTUs	443 ± 21	484 ± 175	380 ± 50	409 ± 18	396 ± 40
Shannon	5.42 ± 0.53	5.05 ± 0.46	4.17 ± 0.98	5.20 ± 0.42	4.51 ± 0.83
Simpson	0.91 ± 0.04	0.89 ± 0.04	0.80 ± 0.13	0.91 ± 0.03	0.81 ± 0.13
Pielou-E	0.62 ± 0.06	0.57 ± 0.05	0.49 ± 0.10	0.60 ±0.05	0.52 ± 0.10

^1^ Mean values ± standard error (SE, n = 3) are presented for each group. Con, control group; HL, high-lipid group; HLA, antibiotic-supplemented high-lipid group; HLB, berberine-supplemented high-lipid group; HLAB, berberine- and antibiotic-supplemented high-lipid group.

## Data Availability

Data supporting this study are available from the corresponding author upon reasonable request.

## Supplementary Materials

The following supporting information can be downloaded at https://www.mdpi.com/article/10.3390/metabo15020118/s1. Figure S1: Correlation analysis (Spearman) of dietary factors and intestinal microbiota at the genus level; Figure S2: Function prediction analysis of gut microbiota by Spearman correlation analysis; Table S1: One-way ANOVA of the top 10 phyla and top 10 genera of microbiota and Firmicutes-to-Bacteroidota ratio in zebrafish under different treatments; Table S2: Significantly changed genera in different groups.
