# Supplementary material for: The Potential Role of Intestinal Microbiota on the Intestine-Protective and Lipid-Lowering Effects of Berberine in Zebrafish (Danio rerio) Under High-Lipid Stress
Source: Metabolites. 2025 Feb 11;15(2):118. doi: 10.3390/metabo15020118 (PMC11857631; doi:10.3390/metabo15020118)
Supplement: Supplementary file 1 [file metabolites-15-00118-s001.zip › metabolites-3438491-supplementary.pdf]

Table S1. One-way ANOVA analysis of the top 10 phylum and top 10 genera of microbiota, and Firmicutes: Bacteroidota ratio in zebrafish of different treatments

	Con	HL	HLA	HLB	HLAB
Phylum					
Proteobacteria	85.35 ± 2.20	59.08 ± 29.01	90.49 ± 1.72	85.51 ± 1.20	82.57 ± 2.32
Firmicutes	4.93 ± 0.37	25.63 ± 18.40	4.38 ± 1.69	6.94 ± 1.45	3.23 ± 0.61
Bacteroidota	1.06 ± 0.20	9.44 ± 7.87	0.89 ± 0.19	1.77 ± 0.57	0.53 ± 0.07
Actinobacteriota	3.22 ± 0.69	1.08 ± 0.42	1.16 ± 0.47	3.43 ± 0.77	2.80 ± 1.03
Planctomycetota	2.07 ± 1.22^{ab}	0.27 ± 0.27^{b}	1.18 ± 1.09^{ab}	0.17 ± 0.16^{b}	5.90 ± 3.24^{a}
Verrucomicrobiota	0.91 ± 0.47	3.43 ± 2.93	0.22 ± 0.13	0.35 ± 0.17	0.98 ± 0.11
Fusobacteriota	1.32 ± 1.04	0.06 ± 0.03	0.72 ± 0.48	0.42 ± 0.33	3.08 ± 2.92
Cyanobacteria	0.58 ± 0.49	0.33 ± 0.23	0.27 ± 0.24	1.05 ± 0.07	0.05 ± 0.04
Patescibacteria	0.16 ± 0.12^{ab}	0.04 ± 0.01^{b}	0.05 ± 0.01^{b}	0.09 ± 0.03^{ab}	0.37 ± 0.19^{a}
Desulfobacterota	0.06 ± 0.03	0.33 ± 0.20	0.11 ± 0.03	0.01 ± 0.00	0.12 ± 0.03
Firmicutes/Bacteroidota	4.82 ± 0.54	4.05 ± 0.93	4.65 ± 0.83	4.65 ± 2.76	6.20 ± 0.95
Genus					
Brevundimonas	15.59 ± 10.43^{ab}	10.25 ± 7.23^{ab}	4.71 ± 3.71^{ab}	24.09 ± 4.08^{a}	1.83 ± 0.55^{b}
Bosea	15.23 ± 7.95	0.45 ± 0.42	2.64 ± 1.56	0.94 ± 0.86	26.42 ± 16.34
Pseudoalteromonas	7.70 ± 2.03	9.59 ± 5.47	10.22 ± 9.11	15.78 ± 1.61	1.60 ± 0.42
Acinetobacter	4.36 ± 1.25	17.06 ± 16.31	2.23 ± 0.86	2.06 ± 0.63	16.97 ± 14.78
Vibrio	7.34 ± 1.41	8.15 ± 4.34	10.01 ± 9.05	15.02 ± 3.23	1.64 ± 0.53
Gemmobacter	6.39 ± 4.17	0.01 ± 0.00	18.43 ± 16.57	1.62 ± 1.61	15.50 ± 7.64
Methylopila	1.46 ± 1.29	0.38 ± 0.37	19.12 ± 18.90	0.01 ± 0.01	2.21 ± 0.78
Ligilactobacillus	0.31 ± 0.01	16.90 ± 16.55	0.17 ± 0.01	0.42 ± 0.23	0.24 ± 0.08
Ralstonia	3.37 ± 1.93^{ab}	2.60 ± 1.90^{b}	1.30 ± 1.00^{b}	8.56 ± 2.32^{a}	0.41 ± 0.10^{b}
Rhodobacter	4.30 ± 3.32	0.06 ± 0.06	7.20 ± 6.65	0.19 ± 0.19	1.55 ± 0.97
Streptococcus	2.20 ± 0.16	2.41 ± 0.96	2.06 ± 1.21	2.34 ± 0.85	1.09 ± 0.06

Values are presented as means ± standard error (SE, n=3). Values with different superscripts in the same row differ significantly ($P < 0.05$). Con, control group; HL,

high lipid group; HLA, antibiotic-supplemented high lipid group; HLB, berberine-supplemented high lipid group; HLAB, berberine and antibiotic-supplemented high lipid group.

Table S2. The significantly changed genus in different groups

	Up	Down
HL VS Con	*Alloprevotella*	*Paracoccus*, *Mesorhizobium*, *Devosia*, *Aminobacter*, *Bacillus*, *Iamia*, *Atopobium*, *Dialister*, *Subdoligranulum*
HLA VS HL	*Cetobacterium*, *Acidovorax*, *HirschiaNK4A214_group*, *Devosia*, *UCG-005*, *Phascolarctobacterium*	*Anaerotruncus*, *Eubacterium_ruminantium_group*, *Rikenella*, *Muribaculaceae_unclassified*, *Akkermansia*, *Alloprevotella*, *Eubacterium_nodatum_group*, *Clostridiales_unclassified*, *Muribaculum*, , *Dubosiella*, *Paramuribaculum*
HLB VS HL	*Pantoea*, *Mesorhizobium*, *Herbaspirillum*, *Stenotrophomonas*, *Nocardioides*, *Acidovorax*, *Candidatus_Arthromitus*, *Pelagibius*, *NK4A214_group*, *Ochrobactrum*, *Candidatus_Methylopumilus*, *Pseudolabrys*, *Barrientosiimonas*, *Acidaminococcus*, *Tagaea*, *UCG-003* , *Saccharospirillum*, *Pannonibacter* , *Dongia*, *Ramlibacter*, *Niastella*, *Acidothermus*, *MND1*, *Steroidobacter*, *Litorivivens*, *Butyricimonas*, *Imperialibacter*, *Frankiales_unclassified*, *Bacillus*, *UCG-005*, *Rhodococcus*, *Prevotella_9* , *Dorea*, *Butyricicoccus*, *Stomatobaculum*, *CAG-56*, *Leifsonia*, *Escherichia-Shigella*, *Faecalibacterium*, *Agathobacter*,	*Muribaculum*, *Odoribacter*, *Anaerotruncus*, *Rikenella*, *Olsenella*, *Intestinimonas*, *Dubosiella*, *Paramuribaculum*, *Erysipelatoclostridium*, *Akkermansia*, *Lachnospiraceae_NK4A136_group*, *Alloprevotella*, *Parabacteroides*, *Desulfovibrio*, *Candidatus-Saccharimonas*, *Prevotella*

	Actinomyces, *Enterococcus*, *Roseburia*, *Lachnoclostridium*, *Christensenellaceae_R-7_group*, *Phascolarctobacterium*, *Dialister* , *UCG-002*, *Clostridium_sensu_stricto_1*, *Subdoligranulum*, *Megasphaera*, *Coprococcus*	
HLAB VS HLA	*Pseudonocardia*, *Gordonia*, *IMCC26207*, *Verrucomicrobium*, *Luteolibacter*, *Actinobacillus*, *Granulicatella*, *Bosea*, *Phreatobacter*, *Methylobacterium-Methylorubrum*, *Devosia*, *Fimbriiglobus*, *Aminobacter*, *Tundrisphaera*, *Paracoccus*, *Haemophilus*, *Mesorhizobium*, *HT002*, *Oscillibacter*	*Christensenellaceae_R-7_group*, *Acidovorax*, *Hirschia*, *Roseburia*, *Megasphaera*, *Escherichia-Shigella*, *Alistipes*, *Turicibacter*
HLAB VS HLB	*Shinella*, *Gemmata*, *Pirellula*, *Fimbriiglobus*, *Tundrisphaera*, *Verrucomicrobium*, *Legionella*, *Comamonas*, *Muribaculum*, *Bdellovibrio*, *Neochlamydia*, *Pseudonocardia*, *Pseudoxanthomonas*, *Odoribacter*, *Gordonia*, *IMCC26207*, *Luteolibacter*, *Anaerotignum*, *Actinobacillus*, *Rhizobium*, *Abiotrophia*, *Methylopila*, *Tabrizicola*, *Paracoccus*, *Staphylococcus*, *Dubosiella*, *Iamia*, *Paramuribaculum*, *HT002*, *Pseudorhodoplanes*, *Oscillibacter*, *Erysipelatoclostridium*, *Campylobacter*, *Bosea*, *Gemmobacter*, *Akkermansia*,	*Agathobacter*, *Herbaspirillum*, *Christensenellaceae_R-7_group*, *UCG-002*, *Xanthomonas*, *Acidovorax*, *Prevotella_9*, *Coprococcus*, *Candidatus_Arthromitus*, *Dorea*, *Pelagibius*, *Ochrobactrum*, *Candidatus_Methylopumilus*, *Pseudolabrys*, *Allobaculum*, *Butyricicoccus*, *Barrientosiimonas*, *Acidaminococcus*, *Tagaea*, *UCG-003*, *Saccharospirillum*, *Pannonibacter*, *Collinsella*, *Arthrobacter*, *Dongia*, *Ramlibacter*, *CAG-56*, *Negativibacillus*, *Niastella*, *Acidothermus*, *MND1*, *Steroidobacter*, *Litorivivens*, *Butyricimonas*, *Imperialibacter*, *Halomonas*, *Bradyrhizobium*, , *Enterococcus*, *Roseburia*, *Lachnoclostridium*,

Microbacterium, *Lachnospiraceae_NK4A136_group*, *Devosia*, *Aminobacter*, *Reyranella*, *Haemophilus*, *Mesorhizobium*, *Desulfovibrio*, *Leptotrichia*, *Gemella*, *Prevotella*	*Dialister*, *Rhodococcus*, *Subdoligranulum*, *Megasphaera*, *Robinsoniella*, *NK4A214_group*, *Lachnospira*, *Brevundimonas*, *Pseudoalteromonas*, *Vibrio*, *Ralstonia*, *Hydrogenophaga*, *Aeromonas*, *Leifsonia*, *Marinomonas Bacteroides*, *Aliidiomarina*, *Shewanella*, *Caulobacter*, *Pseudomonas*, *Escherichia-Shigella*, *Lactobacillus*, *Uruburuella*, *Parabacteroides*, *Faecalibacterium*, *Bifidobacterium*, *Alistipes*, *Actinomyces*, *Ruminococcus*, *Helicobacter*, *Romboutsia*, *Phascolarctobacterium*, *Clostridium_sensu_stricto_1*, *Blautia*, *UCG-005*, *Monoglobus*, *Parasutterella*, *Turicibacter*, *TM7x*

Con, control group; HL, high lipid group; HLA, antibiotic-supplemented high lipid group; HLB, berberine-supplemented high lipid group; HLAB, berberine and antibiotic-supplemented high lipid group.

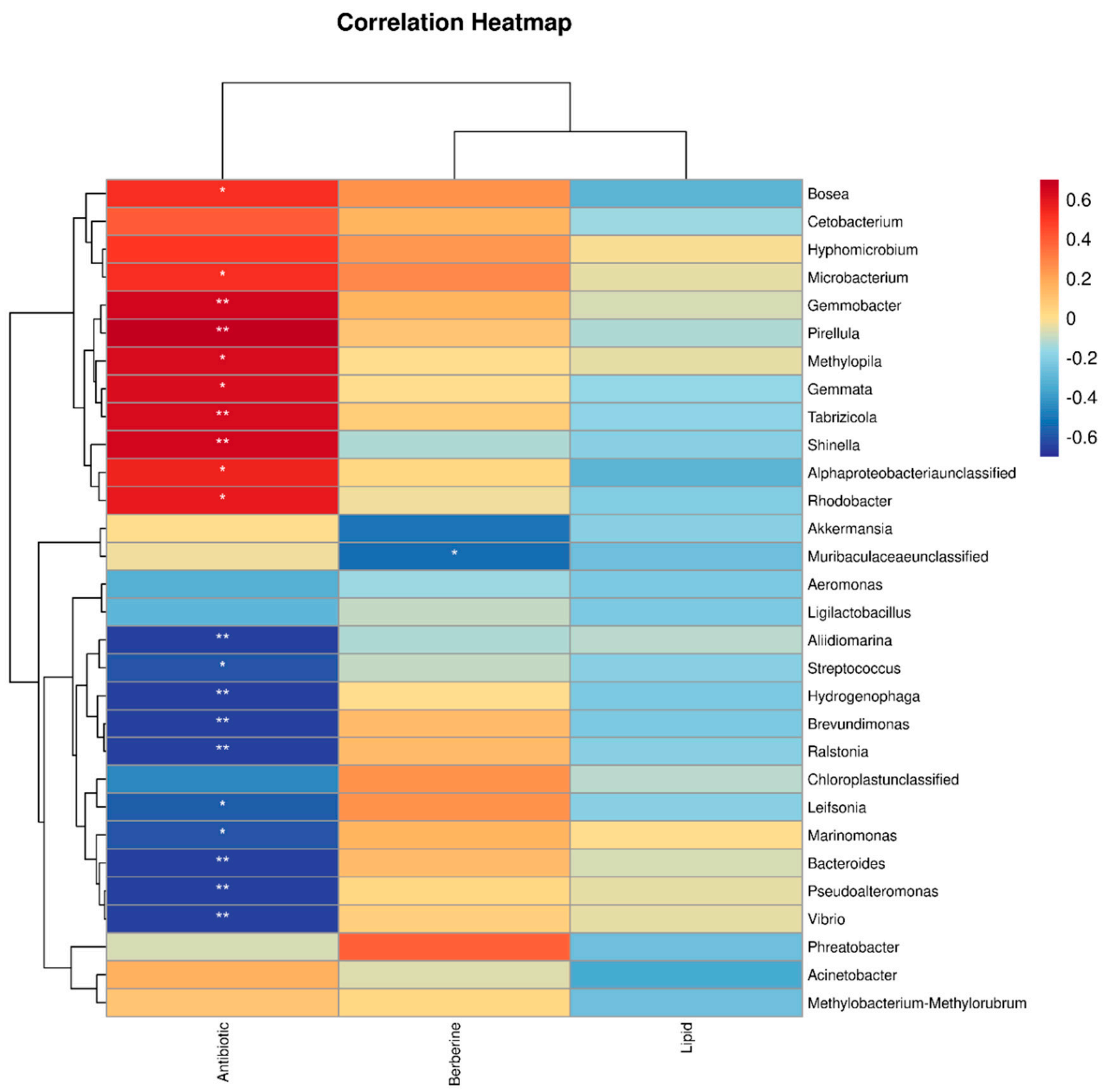

Figure S1. The correlation analysis (Spearman) of dietary factors and intestinal microbiota at the genus level. "*" indicates $P < 0.05$, "**" indicates $P < 0.01$.

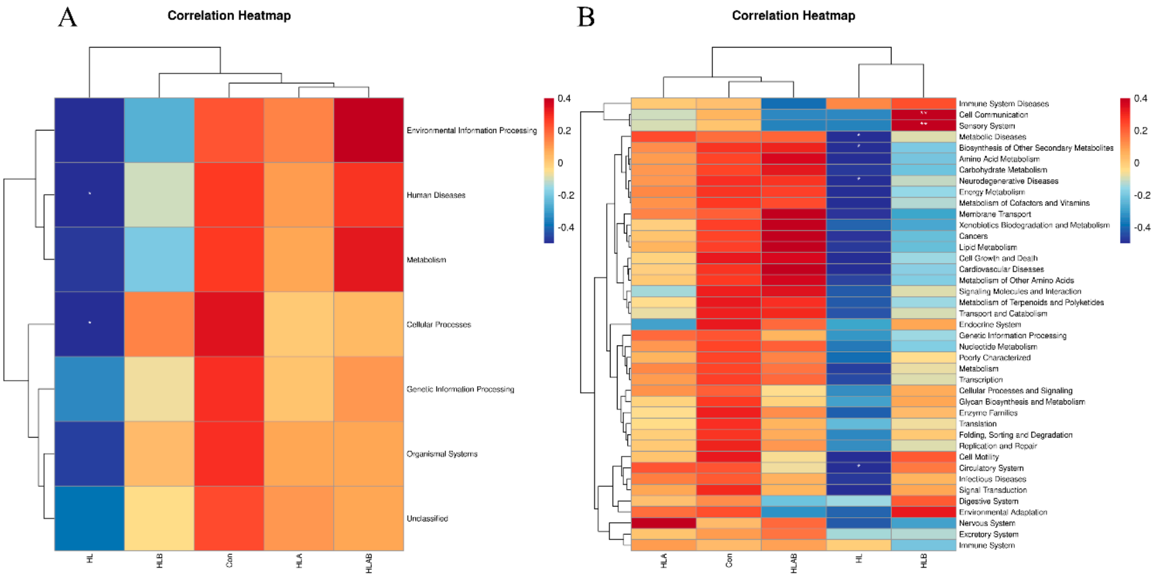

Figure S2. Function prediction analysis of gut microbiota by Spearman correlation analysis. A, Differences in the relative abundance of Level 1 KEGG pathways. B, Differences in the relative abundance of Level 2 KEGG pathways. Con, control group; HL, high lipid group; HLA, antibiotic-supplemented high lipid group; HLB, berberine-supplemented high lipid group; HLAB, berberine and antibiotic-supplemented high lipid group. "*" indicates $P < 0.05$, "**" indicates $P < 0.01$.
